# Adaptation to Endoplasmic Reticulum Stress Enhances Resistance of Oral Cancer Cells to Cisplatin by Up-Regulating Polymerase η and Increasing DNA Repair Efficiency

**DOI:** 10.3390/ijms22010355

**Published:** 2020-12-31

**Authors:** Cho-Yi Chen, Masaoki Kawasumi, Tien-Yun Lan, Chi-Lam Poon, Yi-Sian Lin, Pin-Jou Wu, Yao-Chung Chen, Bing-Hong Chen, Cheng-Hsien Wu, Jeng-Fan Lo, Rueyhung Roc Weng, Yi-Chen Sun, Kai-Feng Hung

**Affiliations:** 1 Institute of Biomedical Informatics, National Yang-Ming University, Taipei 11221, Taiwan; choyichen@ym.edu.tw (C.-Y.C.); zilampoon@gmail.com (C.-L.P.); helen82323@gmail.com (Y.-S.L.); pinjouwu.tw@gmail.com (P.-J.W.); yaochung41@gmail.com (Y.-C.C.); 2 Division of Dermatology, Department of Medicine, University of Washington, Seattle, WA 98109, USA; kawasumi@uw.edu; 3 Department of Medical Research, Division of Translational Research, Taipei Veterans General Hospital, Taipei 11217, Taiwan; s19809040@ym.edu.tw (T.-Y.L.); a30380181@hotmail.com (B.-H.C.); 4 Department of Dentistry, School of Dentistry, National Yang-Ming University, Taipei 11221, Taiwan; wu_ch@vghtpe.gov.tw; 5 Department of Stomatology, Taipei Veterans General Hospital, Taipei 11217, Taiwan; 6 Institute of Oral Biology, National Yang-Ming University, Taipei 11221, Taiwan; jflo@ym.edu.tw; 7 Department of Internal Medicine, National Taiwan University Hospital, Taipei 100225, Taiwan; rh.roc.w@gmail.com; 8 Department of Ophthalmology, Taipei Tzu Chi Hospital, The Buddhist Tzu Chi Medical Foundation, New Taipei City 23142, Taiwan; yichensun.tzuchi@gmail.com

**Keywords:** endoplasmic reticulum (ER) stress response, chemoresistance, cisplatin, polymerase η, DNA repair, damage tolerance

## Abstract

Endoplasmic reticulum (ER) stress response is an adaptive program to cope with cellular stress that disturbs the function and homeostasis of ER, which commonly occurs during cancer progression to late stage. Late-stage cancers, mostly requiring chemotherapy, often develop treatment resistance. Chemoresistance has been linked to ER stress response; however, most of the evidence has come from studies that correlate the expression of stress markers with poor prognosis or demonstrate proapoptosis by the knockdown of stress-responsive genes. Since ER stress in cancers usually persists and is essentially not induced by genetic manipulations, we used low doses of ER stress inducers at levels that allowed cell adaptation to occur in order to investigate the effect of stress response on chemoresistance. We found that prolonged tolerable ER stress promotes mesenchymal–epithelial transition, slows cell-cycle progression, and delays the S-phase exit. Consequently, cisplatin-induced apoptosis was significantly decreased in stress-adapted cells, implying their acquisition of cisplatin resistance. Molecularly, we found that proliferating cell nuclear antigen (PCNA) ubiquitination and the expression of polymerase η, the main polymerase responsible for translesion synthesis across cisplatin-DNA damage, were up-regulated in ER stress-adaptive cells, and their enhanced cisplatin resistance was abrogated by the knockout of polymerase η. We also found that a fraction of p53 in stress-adapted cells was translocated to the nucleus, and that these cells exhibited a significant decline in the level of cisplatin-DNA damage. Consistently, we showed that the nuclear p53 coincided with strong positivity of glucose-related protein 78 (GRP78) on immunostaining of clinical biopsies, and the cisplatin-based chemotherapy was less effective for patients with high levels of ER stress. Taken together, this study uncovers that adaptation to ER stress enhances DNA repair and damage tolerance, with which stressed cells gain resistance to chemotherapeutics.

## 1. Introduction

Endoplasmic reticulum (ER) is the pivotal organelle responsible for multiple cellular functions, including protein folding, synthesis, glycosylation, and trafficking. The homeostasis of ER is often disturbed in cancers by various intrinsic and extrinsic stresses [1]. For example, oncogenic transformation driven by silence of tumor suppressors or by the overproduction of growth factors has been shown to immensely increase protein synthesis, which may cause ER stress when ER functional capacity is overwhelmed [2,3]. Meanwhile, the hostile tumor microenvironment featuring low oxygen, nutrient deprivation, and lactic acidosis also disrupts ER homeostasis, representing an additional source of ER stress in cancers [4]. Because cancers are constantly challenged by numerous cellular stresses, malignant cells utilize diverse strategies to survive these conditions. Sensing and resolving ER stress rely on the integrated response coordinated by three major ER-resident proteins—i.e., the protein kinase R-like endoplasmic reticulum kinase (PERK), activating transcription factor 6 (ATF6), and inositol requiring enzyme 1 (IRE1) [5,6,7,8,9]. These proteins are bound by the chaperone protein (BiP, also known as glucose-related protein 78 (GRP78)) in ER in monomeric inactive forms [10,11]. Upon ER stress, BiP is up-regulated and dissociated from IRE1, PERK, and ATF6, which then trigger downstream signaling pathways to attenuate global protein synthesis and augment the expression of a selected set of genes that are involved in protein folding or degradation, aiming to restore ER homeostasis [4].

In addition to ER stress, chemotherapy represents another source of cellular stress for cancers, and tumor cells often manage to acquire chemoresistance to enhance their survival. The mechanisms underlying chemoresistance involve mutations or differential expression of genes to alter cellular responses [12]. Tumor cells may limit the uptake or boost the efflux of drugs. The chemotherapeutic agents may be detoxified, and the targets of the drug may be modified by tumor cells to increase their chemoresistance [13]. Tumor cells may also increase their capacity to repair and/or tolerate the drug-induced damage [14], or alter the expression of proapoptotic or prosurvival proteins, promote epithelial–mesenchymal transition (EMT) [15], or enter quiescence [16]. Moreover, tumor cells may exploit different cellular responses to acquire chemoresistance.

Since the mode of action varies with chemotherapy, the mechanisms of chemoresistance are directed to each type of drug. Cisplatin is a platinum-based chemotherapeutic agent that cross-links adjacent purines and blocks replicative DNA polymerases to induce apoptosis. Cisplatin resistance has been linked to translesion synthesis (TLS) and nucleotide excision repair (NER) pathways. Specifically, TLS is a DNA damage tolerance process mediated by specialized DNA polymerases, which carry less restricted catalytic sites and thus are capable of replication across certain damage in DNA [17,18,19]. For cisplatin-DNA lesions, the polymerase η with the assistance of monoubiquitinated proliferating cell nuclear antigen (PCNA) has emerged as the main TLS polymerase that allows cancers to develop resistance to this type of chemotherapy [20,21,22]. On the other hand, NER is a versatile DNA repair system for various DNA damages including those induced by platinum-based chemotherapy [23,24]. NER requires the coordination of numerous protein groups to recognize and repair DNA lesions on the genome and transcribed strands [25], and the efficiency of NER is closely associated with chromatin accessibility, damage types, and NER-associated molecules [26,27,28]. 

Among the NER-associated molecules, p53 is the hub of numerous pathways that critically determine the fate of the cell following the induction of DNA damage [29]. The actions of p53 are subjected to multiple regulations, including expressional modulation, post-translational modifications and subcellular localization [30]. Indeed, p53 in the nucleus mediates the transcriptional control of cell cycle and apoptosis [31], and cytoplasmic p53 triggers the cytochrome C release and caspase-3 activation to induce mitochondria-mediated cell death [32,33]. In addition to a role in DNA repair, p53 has recently been implicated in TLS for the survival of UV-irradiated cells [34]. Notably, several studies also connect ER stress response to p53 signaling pathways, showing that ER stress stimulates p53 expression and induces p53 translocation [35,36]. Accordingly, the effect of ER stress on DNA damage response and apoptosis following chemotherapy is possibly associated with the regulation of p53.

Cancer progression is accompanied by the progressive accumulation of cellular stresses at the level tolerable to tumor cells. Clinically, patients with late-stage cancers, often in suboptimal conditions, usually respond poorly to chemotherapy. Because ER stress is a constant and prominent cellular stress in cancers, in this study we investigated whether and how adaptation to ER stress promotes chemoresistance in cancers. A better understanding of the basis upon which stressed cells gain resistance to chemotherapeutics may contribute to an improvement of response to cancer therapy.

## 2. Results

### 2.1. Cancer Cells Adapt to Persistent ER Stress with Phenotypic Transition

**Figure 1:**
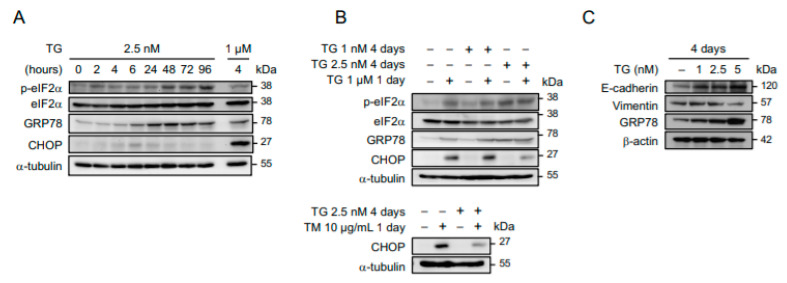
Adaptation to persistent endoplasmic reticulum (ER) stress is accompanied by phenotypic transition. (**A**) OECM1 cells were grown in culture media containing 2.5 nM of thapsigargin (TG) for 0 to 96 h, and time-dependent induction of ER stress response assessed by the levels of phospho-eIF2α, eIF2α, glucose-related protein 78 (GRP78), and C/EBP Homologous Protein (CHOP) were analyzed by Western blots. OECM1 cells treated with 1 µM of thapsigargin for 4 h were used as a positive control to show that ER stress signaling through CHOP in these cells is intact. (**B**) OECM1 cells grown in 1 or 2.5 nM of thapsigargin for 4 days were subsequently mock treated or treated with 1 µM of thapsigargin or 10 µg/mL of tunicamycin (TM) for 1 day, and the induction of ER stress response was analyzed by Western blots. (**C**) OECM1 cells were grown in culture media containing 0, 1, 2.5, or 5 nM of thapsigargin for 4 days, and their mesenchymal–epithelial transition was evaluated by Western blots examining the levels of E-cadherin and Vimentin. The α-tubulin and GAPDH were used as the loading control.

**Figure 2:**
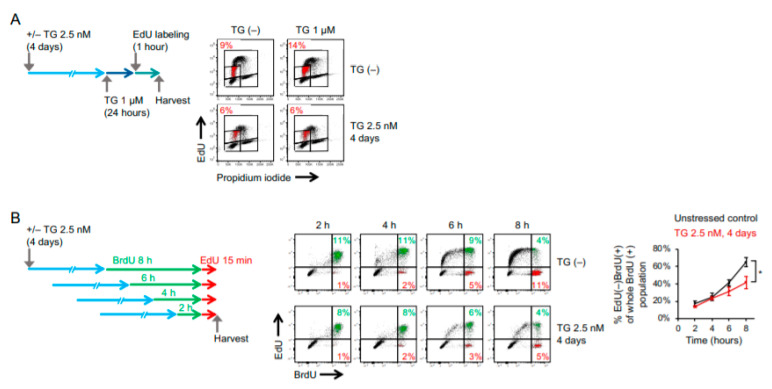
Cancer cells adaptive to ER stress are featured with delayed cell-cycle progression and S-phase exit. (**A**) OECM1 cells with or without 4 days of 2.5 nM thapsigargin pretreatment were treated with 1 µM of thapsigargin for 1 day and subsequently labeled with 5-ethynyl-2-deoxyuridine (EdU) for 1 h. Flow cytometry analysis of cell-cycle subpopulations (G_1_, S, and G_2_ phases) on the basis of EdU incorporation and propidium iodide (PI) staining for DNA content was used to reveal cell population of early-S phase labeled in red. (**B**) OECM1 cells with or without 4 days of 2.5 nM thapsigargin pretreatment were sequentially labeled with 5-bromo-2′-deoxyuridine (BrdU) for 2 to 8 h and EdU for 15 min. The rate of S-phase exit was assessed by the percentage of EdU(−)BrdU(+) of whole BrdU(+) populations measured by flow cytometry analysis. Data from one representative experiment was shown. The mean ± SEM (three independent experiments) of EdU(−)BrdU(+) percentage was plotted. Statistical significance was determined by one-way ANOVA. *, *p* < 0.05.

The ER function of cancers may become progressively impaired under conditions of persistent cellular stress. To characterize the molecular signaling in response to prolonged ER stress, OECM1 and SAS human oral squamous carcinoma cells were maintained in culture media containing 2.5 nM of thapsigargin (a selective inhibitor of ER Ca^2+^-ATPase as an ER stress inducer) for 2 to 96 h, and the levels of ER stress-responsive molecules were examined by Western blot analysis. Here, 2.5 nM of thapsigargin was used because exposure to a higher concentration for an extended period of time was intolerable to these cells and would cause cell death. As shown in Figure 1A, the phosphorylated eIF2α (Ser51) and glucose-related protein 78 (GRP78), two master regulators of ER stress response, in cells grown in a low-dose ER stress inducer were time-dependently up-regulated and sustained at high levels. The induction of C/EBP Homologous Protein (CHOP), which is a downstream target of the PERK-eIF2α-ATF4 axis, peaked 6 h after thapsigargin treatment and gradually returned to the level of the untreated control, implying that cells became adaptive to ER stress. To further demonstrate the ER stress adaptation, OECM1 cells that had been maintained in 1 or 2.5 nM of thapsigargin-containing media for 4 days were subsequently treated with high doses of ER stress inducers (1 µM of thapsigargin or 10 µg/mL of tunicamycin). We found that phospho-eIF2α and GRP78 were significantly up-regulated in cells that had been treated with thapsigargin at a concentration level of above 1 nM. Notably, while 1 µM of thapsigargin or 10 µg/mL of tunicamycin robustly up-regulated CHOP regardless of whether cells were pretreated with 1 nM of thapsigargin, the induction of CHOP was significantly attenuated by a pretreatment of 2.5 nM of thapsigargin (Figure 1B), suggesting that cells became less sensitive to ER stress after a prolonged exposure to 2.5 nM of thapsigargin. Phenotypically, these stress-adaptive cells adopted an elongated morphology and grew slowly. Further characterization of this phenotypic transition revealed an increase in E-cadherin and a decrease in Vimentin expression, thus implying a mesenchymal–epithelial transition (Figure 1C). In addition, the flow cytometry-based analysis of cells labeled with 5-ethynyl-2-deoxyuridine (EdU) and propidium iodide showed that the induction of ER stress led to the accumulation of cells in early-S phase (from 9 to 14%), and this effect was abolished when cells had adapted to prolonged stress (Figure 2A). Importantly, the EdU(+) subpopulation decreased in cells maintained in low-dose ER stress inducers, indicating that stress adaptation was accompanied by a delayed cell-cycle progression. We also examined the progression of the S phase by sequential labeling of cells with 5-bromo-2′-deoxyuridine (BrdU) for 2 to 8 h and subsequently with EdU for 15 min. We found that the efficiency of cells to exit the S phase, as indicated by the percentage of EdU(−)BrdU(+) over the whole BrdU(+) subpopulation, was lowered in cells that had been maintained in 2.5 nM of thapsigargin (Figure 2B). Taken together, these results suggest that cancer cells exhibiting phenotypic transition are able to tolerate prolonged ER stress.

### 2.2. Cancer Cells Adaptive to ER Stress Are More Resistant to Cisplatin-Induced Cytotoxicity

**Figure 3:**
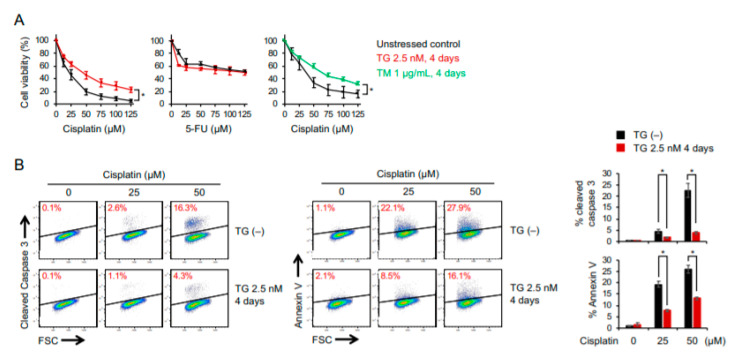
Adaptation to persistent ER stress enhances chemoresistance to cisplatin. OECM1 cells with or without pretreatment of 2.5 nM of thapsigargin or 1 µg/mL of tunicamycin for 4 days were treated with various dose of cisplatin or fluorouracil (5-FU). (**A**) WST-8 assay of dose-dependent cell viability. (**B**) Representative flow cytometry analysis of cell apoptosis by cleaved Caspase 3 or Annexin-V staining. Data from three independent experiments are shown as the mean ± SEM. Statistical significance was determined by one-way ANOVA and Student’s *t*-test. *, *p* < 0.05.

Since the response to chemotherapy is believed to be associated with epithelial–mesenchymal transition, which, as shown in Figure 1C, was reversed by prolonged induction of ER stress, it is critical to investigate how ER stress-adaptive cells respond to chemotherapy. To this end, OECM1 and SAS cells that were maintained in low doses of ER stress inducers (2.5 nM of thapsigargin or 1 µg/mL of tunicamycin for 4 days) were treated with cisplatin or fluorouracil (5-FU) for 24 h, and dose-dependent cytotoxicity was determined. We found that the adaptation to ER stress endowed cancer cells with increased resistance to cisplatin but not 5-FU (Figure 3A). An increase in cisplatin resistance was also observed in the SAS and HSC-3 cancer cell lines (Supplementary Figure S1). Through the detection of cleaved caspase 3 and Annexin-V, we showed that the population of apoptotic cells was significantly decreased in cells pretreated with a low dose of thapsigargin (Figure 3B). 

### 2.3. RNA Sequencing Analysis Reveals a Global Suppression of DNA Replication and Cell-Cycle Progression in ER Stress-Adaptive Cells

**Figure 4:**
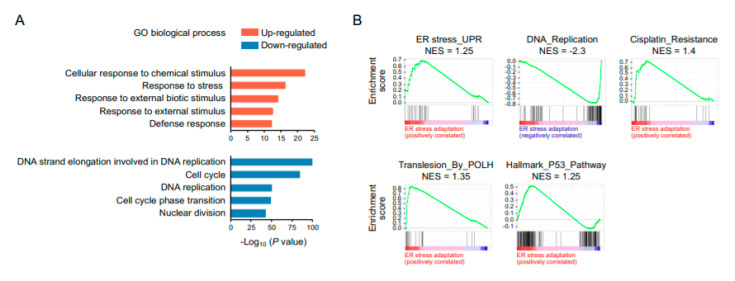
RNA sequencing analysis reveals a global suppression of DNA replication and cell-cycle progression in ER stress-adaptive cells. (**A**) Gene ontology analysis of biological processes that are enriched in up-regulated and down-regulated genes. (**B**) Gene Set Enrichment Analysis (GSEA) of over-represented and under-represented gene sets. NES, normalized enrichment score.

To probe the mechanisms by which cells adaptive to ER stress gain resistance to cisplatin, we used RNA sequencing to profile gene expression in ER stress-adapted cells, aiming to identify molecular signatures relevant to chemoresistance. The gene ontology (GO) analysis revealed that the up-regulated genes were enriched for GO terms related to biological processes of cellular response to a chemical stimulus, response to stress, response to external biotic stimulus, and defense response, whereas the down-regulated genes were related to DNA strand elongation, cell cycle, DNA replication, cell-cycle phase transition, and the nuclear division process (Figure 4A). The Gene Set Enrichment Analysis (GSEA) showed an over-representation of gene sets associated with ER stress response and cisplatin resistance and an under-representation of gene sets associated DNA replication (Figure 4B). Because the mechanism of action of cisplatin is mediated by binding to DNA and interfering with DNA replication and repair, cisplatin resistance has been attributed to the efficient removal of DNA lesions and prompt rescue of stalled replication, primarily by nucleotide excision repair (NER) and translesion synthesis, respectively. Therefore, the gene sets linked to these processes were our focus of attention. Consequently, GSEA further identified that the gene sets involved in *POLH*-mediated translesion synthesis and p53 pathway were over-represented in cohorts of ER stress adaptation.

### 2.4. POLH-Encoded Polymerase η Is Up-Regulated by Prolonged ER Stress and Contributes to Cisplatin Resistance

**Figure 5:**
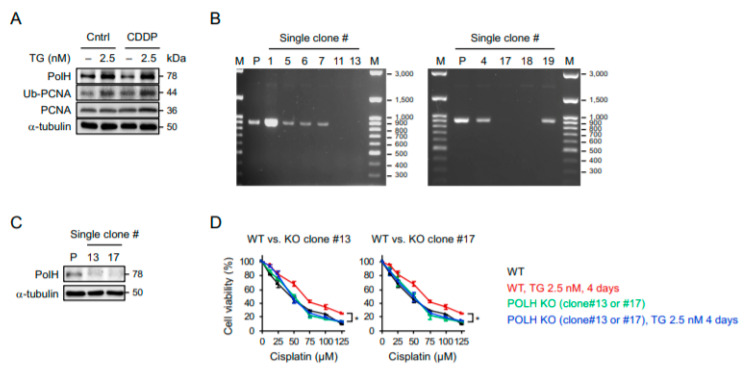
Polymerase η is up-regulated in ER stress-adapted cells and is required for their acquisition of cisplatin resistance. (**A**) OECM1 cells with or without 4 days of 2.5 nM thapsigargin pretreatment were treated with 100 µM of cisplatin for 1 day, and the expression of polymerase η, proliferating cell nuclear antigen (PCNA), and ubiquitinated PCNA (Ub-PCNA) were examined by Western blots. The α-tubulin was used as the loading control. (**B**) PCR analysis of single-cell clones following clustered, regularly interspaced, short palindromic repeat RNA-guided (CRISPR)-based knockout of POLH using primers designed to amplify the region flanked by sgRNA docking sites. (**C**) Western blot analysis of the expression of polymerase η in two identified POLH-knockout clones. (**D**) WST-8 assay of cell viability after treatment with various doses of cisplatin. Data from three independent experiments are shown as the mean ± SEM. Statistical significance was determined by one-way ANOVA. *, *p* < 0.05.

Since polymerase η encoded by *POLH* has been linked to cisplatin resistance [20,21,22], we sought to determine whether ER stress-induced cisplatin resistance is indeed mediated by polymerase η. As show in Figure 5A, the levels of polymerase η and the ubiquitination of proliferating cell nuclear antigen (PCNA) (a processivity factor for DNA polymerase’s recruitment upon ubiquitination) were increased by prolonged ER stress. Notably, the cisplatin alone had no inductive effect on polymerase η or the ubiquitination of PCNA. To further investigate the role of polymerase η in cisplatin resistance, we created *POLH*-knockout cells by the employing clustered, regularly interspaced, short palindromic repeat RNA-guided (CRISPR)/Cas9 system with two separated sgRNAs targeting the sequences before exon 2 and at the end of exon 3 of the *POLH* to delete a gene segment containing an ATG start codon. Using primers designed to amplify the region flanked by these two sgRNA docking sites, the PCR only amplified the wild-type allele (916 bp). As a result, the single-cell clones (#11, 13, 17, and 18) with deletions of *POLH* were identified by a complete lack of PCR products (Figure 5B), and the subsequent Western blot analysis further revealed minimal expression of the polymerases η in two *POLH*-knockout clones (Figure 5C). Importantly, while the wild-type cells became more resistant to cisplatin after a prolonged pretreatment with the ER stress inducer, the *POLH*-knockout cells with and without the induction of ER stress exhibited similar sensitivities to cisplatin (Figure 5D), suggesting that the protective effect of adaptive ER stress response on cisplatin-induced cytotoxicity was abrogated by the knockout of polymerase η. Since DNA polymerases are mostly active during DNA synthesis, we used EdU to label replicating cells and examined whether the protective effect is induced mainly in S-phase cells. Intriguingly, a reduction in cleaved Caspase 3 (+) population was observed not only in EdU-labeled cells but also EdU-unlabeled cells (Supplementary Figure S2). Accordingly, the protection from cisplatin cytotoxicity is not restricted to the replicating cells, and additional mechanisms are likely involved in the ER stress-mediated cisplatin resistance.

### 2.5. Adaptation to ER Stress Promotes p53 Nuclear Translocation and Limited the Accumulation of Cisplatin-DNA Lesions

**Figure 6:**
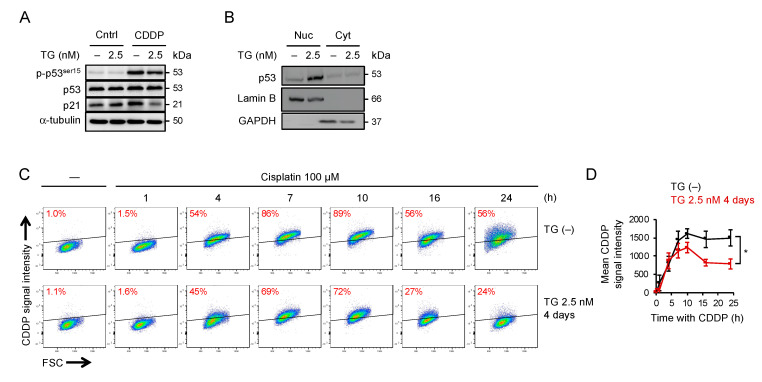
Adaptation to prolonged ER stress enhance p53–p21 prosurvival axis and decreases accumulation of cisplatin-induced DNA damage. OECM1 cells with or without 4 days of 2.5 nM thapsigargin pretreatment were treated with 100 µM of cisplatin for 1 day, and (**A**) the expression of phosphorylated p53 at Ser51, p53, and p21, (**B**) nuclear vs. cytoplasmic fraction of p53, and (**C**) chromatin-bound vs. nuclear fraction of p53, were examined by Western blots. The α-tubulin, Lamin B, GAPDH, and Histone H3 were used as the loading control. (**D**) Representative flow cytometry analysis of the levels of cisplatin-DNA lesions 1, 4, 7, 10, 16, and 24 h after cisplatin treatment. Data from three independent experiments are shown as the mean ± SEM. Statistical significance was determined by one-way ANOVA. *, *p* < 0.05.

Because persistent ER stress causes global translational repression, we speculated that the depletion of key molecules that trigger apoptosis is another mechanism responsible for cisplatin resistance. The central role of p53 in apoptosis, DNA repair, and cell-cycle arrest is well known, and a recent study also showed that p53 has a predominant role in translesion synthesis [34]. Therefore, as supported by our RNA sequencing analysis, we examined the response of p53 to persistent ER stress. As shown in Figure 6A, the level of p53 and its phosphorylation at Ser-15 were unaffected by a prolonged treatment with a low dose of thapsigargin, suggesting that the ER stress at this level allowed adaptation and would not cause DNA damage. The level of p21, a downstream effector of p53, appeared to be up-regulated in ER stress-adaptive cells, although the extent of increase was less significant. In addition, we found that the p53 phosphorylation and the induction of *PUMA* and *NOXA*, two major proapoptotic transcriptional targets of p53, by cisplatin were comparable between control and stress-adaptive cells, suggesting that p53 signaling remained intact in cells adaptive to ER stress (Supplementary Figure S3). Notably, while the overall expression of p53 was unchanged, the Western blot analysis of subcellular fractionated samples revealed that a fraction of p53 in stress-adaptive cells was translocated to nucleus (Figure 6B) along with nuclear translocation of 53BP1 (Supplementary Figure S4). Meanwhile, we used the antibody that specifically recognizes cisplatin-induced DNA lesions with flow cytometry and found that treatment of cells with cisplatin resulted in a time-dependent incorporation of cisplatin-DNA lesions, which peaked approximately 10 h after initial treatment, followed by a gradual decline in lesions presumably by activation of DNA repair (Figure 6C). Remarkably, the accumulation of cisplatin-DNA lesions was reduced by a large amount in cells that had been pretreated with the ER stress inducer (Figure 6D). Consistently, the level of DNA damage assessed by phospho-H2AX after cisplatin treatment was lower in stress-adaptive cells than that in control cells (Supplementary Figure S5). Using fluorophore-conjugated cisplatin [37], we found that the uptake of drugs was not affected in stress-adaptive cells (Supplementary Figure S6). We also found that the level of GADD45, which has been implicated in DNA repair and cell-cycle control [38], was up-regulated in stress-adaptive cells (Supplementary Figure S7). Because ER stress and oxidative stress are closely linked in the events of cell homeostasis and apoptosis [39,40], we also examined the oxidative burden in stress-adaptive cells using MitoSOX. As shown in Supplementary Figure S8, the stress-adaptive cells were in a more reduced state; however, the levels of mitochondrial reactive oxygen species (ROS) in these cells following cisplatin treatment were not significantly decreased compared to the unstressed cells. Interestingly, p53-knockdown OECM1 or HSC-3 cells were unable to tolerate prolonged ER stress and were rarely viable after cisplatin treatment (Supplementary Figure S9). Taken together, our findings suggest that adaptation to ER stress led to nuclear translocation of p53 and equipped cells with a superior ability to limit the cisplatin-DNA damage.

### 2.6. Cispltain-Based Chemotherapy May Be Less Effective for Patients with Oral Cancers Exhibiting High Levels of ER Stress

**Figure 7:**
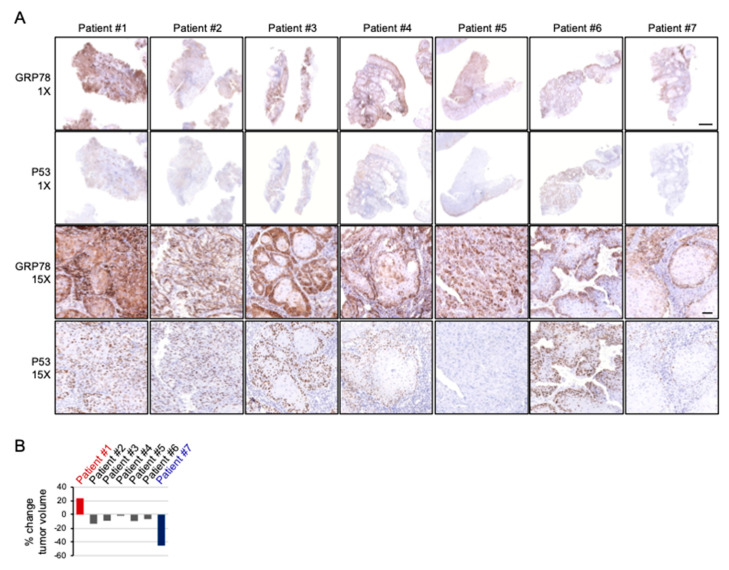
The pattern and intensity of GRP78 positivity coincides with nuclear p53 on immunostaining and is associated with the response to cisplatin-based chemotherapy. (**A**) Whole biopsies and representative GRP78 and p53 immunostaining of specimens of oral cancers taken before cisplatin-based induction chemotherapy. Dominant p53 nuclear staining coincides with the area of strong GRP78 positivity except for patient #5. (**B**) The percentage of relative tumor volume change of patients after two courses of induction chemotherapy. Patient #1 shows progression (23%) and patient #7 shows regression (45%) markedly after treatment. Scale bar: 2 and 0.1 mm for 1× and 15×, respectively.

Moving beyond the in vitro model, to further evaluate the response to cisplatin-based chemotherapy of cancers sustaining ER stress, the clinical biopsies from seven patients were immunostained for GRP78 and the change in gross tumor volumes were assessed by comparing the radiographic imaging prior to initiation of chemotherapy and a direct measurement of the surgical specimen following treatment. The biopsies were taken before treatment commenced; therefore, the change in tumor volume can be used to evaluate how the intrinsic ER stress in cancers affects their response to cisplatin-based chemotherapy. As shown in Figure 7A, oral cancer biopsies exhibited various GRP78 immunointensities and patterns, with strong homogeneous positivity in almost all tumor cells by patient #1, as well as relatively weak positivity limited to peripheral tumor area by patient #7. Remarkably, after induction chemotherapy, the tumor of patient #1 progressed and the tumor of patient #7 regressed markedly (23% increased and 45% decreased, respectively), whereas the majority of patients (5 out of 7) showed a modest response (Figure 7B). Interestingly, except for patient #5, immunostaining of nuclear p53 nearly completely followed the staining pattern of GRP78, which was consistent with our findings with cells in vitro. Sequencing *TP53* exons from tumor DNA found mutations in all seven patients (Table 1).

## 3. Discussion

ER stress response is generally regarded as an adaptive survival mechanism and has been implicated in the acquisition of chemoresistance [41,42,43]. Indeed, it has been shown that the levels of numerous ER stress markers are correlated with poor response to various chemotherapeutics in a variety of cancers and cell lines, and that pharmacological or genetic inhibition of ER stress signaling increase the susceptibility to chemotherapy [42,43,44,45,46]. Notably, most of the evidence came from studies that correlated the expression of ER stress markers with poor prognosis or that demonstrated proapoptosis following the knockdown of ER stress-responsive genes. To our knowledge, only a few studies (including the present one) have demonstrated an increase in chemoresistance in cancers by inducing ER stress rather than by genetic manipulations [43]. However, such demonstrations are important because ER stress accumulation in cancers is essentially not caused by genetic modifications. Moreover, ER stress response consists of coordinated transcriptional regulatory networks and may have context-dependent or opposite effects on cells. In fact, signaling through PERK, IRE1, and ATF6 can trigger cell apoptosis [47], and it has also been shown that moderate and high ER stress generate distinct patterns of stress signaling and apoptosis activation. Accordingly, we used a nongene targeting approach to allow cancer cells to become adaptive to mild and prolonged ER stress. Our findings, consistent with those of a study using cells that had undergone stepwise selection under high ER stress conditions [43], indeed support that the proposition that adaptive ER stress response enables the acquisition of chemoresistance.

To date, several mechanisms associated with ER stress signaling for acquiring chemoresistance have been reported. For example, the PERK-mediated signaling via eIF2α-ATF4 and Nrf2 is shown to up-regulate the MDR-related protein 1 [43], detoxifying enzymes [48], or autophagy [49] for promoting cell survival under hypoxia and chemotherapy [50]. Meanwhile, enhanced IRE1 activity has been demonstrated to increase the production of protumorigenic cytokines in breast cancer cells to survive paclitaxel [51], and ATF6 is required to promote mTOR activation and STAT3 signaling to gain chemoresistance [52,53,54]. GRP78 is also found to confer resistance to etoposide and estrogen starvation by blocking BIK- and NOXA-induced caspase-dependent apoptosis [55,56]. However, since we showed that most ER stress signaling during adaptation was declining and became insensitive to further challenge, it is plausible that other mechanisms beyond these pathways may be responsible for the acquisition of chemoresistance in adaptive cells. Phenotypically, we found that ER stress-adaptive cells grow slowly and are featured with a prolonged S phase and a delayed cell-cycle progression. Interestingly, growth arrest and cellular quiescence or dormancy have been recognized as a key step to developing therapy resistance [16,57,58,59]. Previous studies also suggested a functional link between ER stress response and tumor dormancy, which was supported by the findings that PERK activation inhibited cyclin D1 synthesis for G1 arrest, and that ATF6 was constitutively active in dormant cells [54,60]. Collectively, although further investigation is needed, it is reasonable to speculate that the reduction in replication rate might be one of the stress-adaptive mechanisms for cancers to gain chemoresistance. 

While the chemoresistance in ER stress-adaptive cells can be related to the reduction in replication and cell-cycle progression, our findings showing preferential resistance to cisplatin, but not 5-FU, imply the involvement of a more specific mechanism. Polymerase η has been characterized as a specialized DNA polymerase capable of replicating across ultraviolet- or cisplatin-induced DNA damage to prevent cell-cycle arrest [61,62,63,64,65,66], and a high level of polymerase η expression is correlated with poor prognosis among patients with platinum-based treatment of multiple cancers [66,67]. Accordingly, we found that the levels of polymerase η and PCNA ubiquitination are increased in ER stress-adaptive cells, implying that the ability of these cells to survive cisplatin treatment is improved. Additionally, knockout of polymerase η abrogated the effect of ER stress induction on cells surviving cisplatin, further suggesting that the acquired cisplatin resistance is at least partly mediated by polymerase η. The cellular activity of polymerase η is critically regulated by its relocation promoted by PCNA ubiquitination following DNA damaging stimuli, such as UV irradiation, nucleotide deprivation, or chemotherapy. However, since DNA damage is not induced in ER stress-adaptive cells, it remains unclear how the activity of polymerase η in stress-adaptive cells is promoted. Notably, several recent studies uncovered that PCNA was ubiquitinated even in the absence of DNA damage to recruit translesion synthesis polymerases for completion of DNA replication [68,69,70]. Thus, delayed progression of S phase and cell-cycle transition in stress adaptive cells can serve as the contributing factors to the increased activity of polymerase η, consequently accounting for the enhanced resistance of these cells to cisplatin treatment.

Intriguingly, the cisplatin resistance is not preferentially induced in the S phase, given the fact that the function of polymerase η is mostly related to DNA replication. In addition, even though the recruitment of polymerase η can occur outside the S phase [71,72,73], whether these polymerases remain active is uncertain. Interestingly, we found that p53 in ER stress-adaptive cells preferentially relocates to nucleus. Because p53 plays an important role in DNA repair and *POLH*-mediated translesion synthesis [74], the ER stress-induced cisplatin resistance is likely mediated by its effect on p53 nuclear translocation to promote these two cellular processes. Thus far, while the ER stress response has been implicated in the regulation and translocation of p53 [35,36,75], both a promotive and an inhibitory role of ER stress in DNA repair have been reported [76,77,78,79]. Nonetheless, since we showed that the decline in the level of cisplatin-DNA damage by DNA repair is more significant in ER stress-adaptive cells, it is plausible that stress adaptation induces p53 and its nuclear distribution to promote DNA damage tolerance and repair. Importantly, this idea is supported by previous studies showing that a pretreatment with low doses of UV or ionizing radiation (IR) protects cells from subsequent UV or IR exposures in a p53-dependent manner [34,80]. Here, our study extends this idea by demonstrating that the protective effect can be promoted by ER stress, a type of non-DNA damaging stress, to increase cell survival upon cisplatin-DNA damage.

Notably, because immortalized cancer cell lines were used in this study, this raises a concern that the ER stress-induced cisplatin resistance might be attributed to p53 mutations. OECM1 cells harbor the V173L missense mutation in the DNA binding domain of p53 [81]. Nonetheless, as shown in this study, the ability to maintain proper cell-cycle checkpoints and transactivate proapoptotic genes seems to be unaffected in this cell type, thus arguing that the protective effect of adaptive ER stress response is not a consequence of p53 mutation. Moreover, an increase in resistance to cisplatin upon stress adaptation was also observed in HSC-3 and SAS cells, which carry distinct recessive mutations and are considered to have functional p53 [82]. In addition, all patients in this study, including six patients who exhibited p53 nuclear staining in the area of strong GRP78 positivity and of patient who did not, harbor p53 mutations, suggesting that our findings are less likely biased by p53 mutations. Further supporting a role of p53 in ER stress-mediated cell survival, we found that knockdown of p53 in OECM1 or HSC-3 cells sensitized these cells to ER stress and made them rarely viable following cisplatin treatment.

While our study observed a significant reduction in cisplatin-DNA lesions in stress-adaptive cells, the DNA repair or tolerance may not be the only mechanism accounting for their cisplatin resistance. ER stress and oxidative stress have been closely linked—the oxidative protein folding in ER generates ROS as a byproduct, which can disturb protein folding to cause ER stress. Meanwhile, reducing disulfide bonds of misfolded proteins to resolve ER stress depletes glutathione, leading to the accumulation of ROS and compromised ER redox balance [39,83]. On the other hand, activation of PERK by ER stress induces ATF4 and Nrf2 to transactivate several antioxidant genes, and the ER stress response is shown to promote the adaptation to ROS production [84]. Accordingly, as ROS generation has been identified as a crucial mediator of cisplatin cytotoxicity [85], the resistance to cisplatin may be a consequence of ER stress-mediated ROS adaptation. However, we found that the level of ROS after cisplatin treatment was not significantly decreased in stress-adaptive cells, even though these cells were in a more reduced state before treatment. Possibly, the cells that have been adapted to ER stress become less responsive to cisplatin-induced ROS, which would otherwise have activated the PERK-ATF4- or Nrf2-mediated antioxidant activities.

Although the removal of cisplatin-DNA lesions is shown to be more efficient in cells adaptive to ER stress, this study is limited to identifying the mechanism by which DNA repair is improved. Indeed, our RNA sequencing data did not reveal an up-regulation of genes associated with nucleotide excision repair, leading to the assumption that enhanced DNA repair is a consequence of other relevant biological processes. In this regard, an attribute of delayed replication and cell-cycle progression during damage response is to allow times for DNA repair [86], and the accelerated replication progression instead induces the accumulation of DNA damage [87]. Meanwhile, the contribution of the efflux pump to acquired chemoresistance in ER stress-adaptive cells seems to be less substantial because these cells show almost identical kinetics of the cisplatin-DNA lesion accumulation (from 0 to 7 h after cisplatin treatment) by flow cytometry, comparable fluorescent intensities from the uptake of fluorophore-conjugated cisplatin, as well as similar expressions of pump-encoding genes on RNA sequencing analysis.

**Figure 8:**
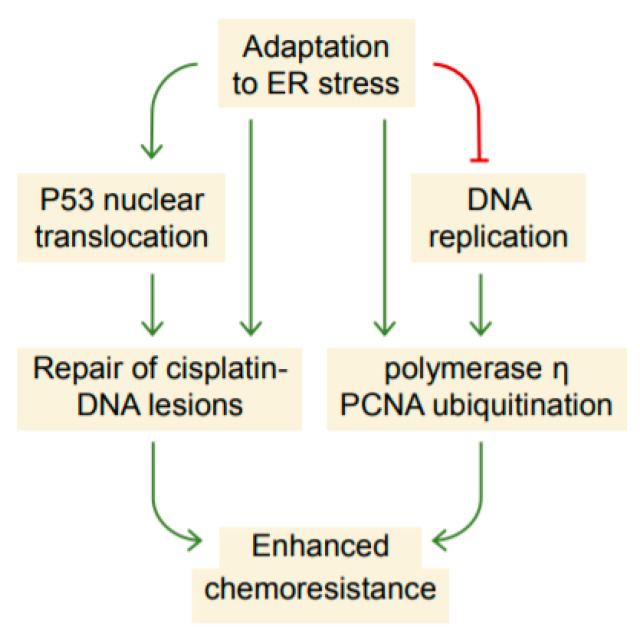
Proposed schematic depicting the enhancement of chemoresistance as a consequence of adaptation to ER stress. The adaptive ER stress response is accompanied by a suppression of DNA replication and cell-cycle progression that, to some extent, resembling the response to replication stress. As a result, cells promote the expression of polymerase η and nuclear translocation of p53, which may possibly increase the efficiency of translesion synthesis and DNA repair, leading to enhanced chemoresistance.

In summary, our study uncovers that the adaptation to ER stress accompanies a coordinated mechanism involving the suppression of DNA replication, up-regulation of polymerase η, and nuclear translocation of p53, which subsequently limit the accumulation of cisplatin-DNA lesions likely by DNA damage tolerance and repair, leading to increased cisplatin resistance (Figure 8). Our results also imply that chemoresistance and EMT or mesenchymal-epithelial transition (MET) can be functionally uncoupled. Because cellular stress is increased as cancers progress to late-stage for which chemotherapy is often indicated, future studies dissecting in great detail how the cellular stress response mitigates chemotherapy-induced damage will lead to the development of therapeutic strategies to overcome the chemoresistance.

## 4. Materials and Methods

### 4.1. Cell Lines and Culture Conditions

The OECM1 and SAS cancer cell lines derived from squamous cell carcinoma of a gingival and tongue cancer patient [88], respectively, were grown in Roswell Park Memorial Institute Medium (RPMI; #11-100, Biological Industries, Beit Haemek, Israel) and Dulbecco’s Modified Eagle Medium (DMEM; #11-0550, Biological Industries, Beit Haemek, Israel) supplemented with 10% Fetal Bovine Serum (FBS; #10438-028, Thermo Fisher Scientific, Waltham, MA, USA), 1% penicillin-streptomycin (#15140-122, Thermo Fisher Scientific, Waltham, MA, USA), and 1% glutamine. For the induction of ER stress at a level allowing adaptation, 5 × 10^4^ cells in culture media containing 1 or 2.5 nM of thapsigargin or 1 µg/mL of tunicamycin were plated in a 6 cm dish and were harvested at designated times for Western blot analysis or subjected to subsequent experiments. For treatment of high-dose ER stress inducers or chemotherapeutic agents (cisplatin or 5-FU), thapsigargin or tunicamycin-containing cell culture media were aspirated and cells were washed with a warm phosphate-buffered saline (PBS), followed by the addition of cell culture media containing 1 µM of thapsigargin, 10 µg/mL of tunicamycin, cisplatin (up to 125 µM), or 5-FU (up to 125 µM). The cell viability was then analyzed by WST8 or flow cytometry assays.

### 4.2. Patients and Tissue Samples

This study was approved by the IRB (IRB-TPEVGH No. 2017-12-015BCF#1) and written informed consent was obtained from all patients. This study included 7 male patients who were newly diagnosed with squamous cell carcinoma of oral cavity. The biopsies and radiographic imaging were taken before the initiation of any treatment. All patients received two courses of cisplatin-based induction chemotherapy (the combination of Taxotere, Platinol, and Fluorouracil, so-called TPF regimen), followed by surgical treatment. 

### 4.3. Western Blot

Cells were lysed in cell lysis buffer (9803S, Cell signaling, Danvers, MA, USA) containing PhosSTOP mini (04906837001, Sigma-Aldrich, St. Louis, MI, USA) and a protease inhibitor cocktail (04693124001, Sigma-Aldrich, St. Louis, MI, USA). Protein concentration was measured using the protein BCA assay kit (23227, Thermo Fisher Scientific, Waltham, MA, USA) and equal amounts of protein were boiled in NuPAGE 4X LDS Sample buffer (NP0007, Thermo Fisher Scientific, Waltham, MA, USA), separated by 15% SDS/PAGE, and transferred onto a nitrocellulose membrane (10484060, Bio-Rad, Hercules, FL, USA). The membrane was incubated for 1 h in blocking buffer (Tris-buffered saline with 0.1% Tween (TBS-T) and 5% nonfat dry milk) and then probed by overnight incubation at 4 °C with the following primary antibodies: anti-phospho-eIF2α (3597, Cell Signaling, Danvers, MA, USA), anti-eIF2α (9722, Cell Signaling, Danvers, MA, USA), anti-GRP78 (C50B12, Cell Signaling, Danvers, MA, USA), anti-CHOP (L63F7, Cell Signaling, Danvers, MA, USA), anti-Vimentin (550513, BD Pharmingen, San Jose, CA, USA), anti-PolH (A301-231A, Bethyl, Montgomery, AL, USA), anti-PCNA (ab29, Abcam, Cambridge, MA, USA), anti-Ubiquityl-PCNA (13439, Cell Signaling, Danvers, MA, USA), anti-p53 (9282, Cell Signaling, Danvers, MA, USA), anti-phospho-p53^ser15^ (9284, Cell Signaling, Danvers, MA, USA), anti-p21 (ab109520, Abcam, Cambridge, MA, USA), anti-Lamin B (ab16048, Abcam, Cambridge, MA, USA), anti-Histone H3 (GTX122148, GeneTex, Irvine, CA, USA), anti-E-cadherin (610182, BD Pharmingen, San Jose, CA, USA), anti-GADD45 (180768, Abcam, Cambridge, MA, USA), anti-α-tubulin (SC-5286, Santa Cruz, Dallas, TX, USA), anti-β-action (MA5-15739, Thermo Fisher Scientific, Waltham, MA, USA), or anti-GAPDH (MA1-16757, Thermo Fisher Scientific, Waltham, MA, USA) antibodies. After washing in TBS-T, the blot was incubated with horseradish peroxidase-conjugated secondary antibodies and detection was performed using the enhanced chemiluminescence system (WBKLS0500, Millipore, Burlington, VT, USA) as described by the manufacturer. All Western blot analyses were performed separately in each of the three independent experiments.

### 4.4. Flow Cytometry Analyses

For experiments evaluating the progression of cell-cycle phases upon adaptation to ER stress, cells that had been maintained in low-dose ER stress inducers for 4 days were washed with a warm PBS with or without subsequent incubation in 1 µM of thapsigargin for 1 day, and were then incubated with media that contained 15 µM of EdU at 37 °C for 1 h. For experiments evaluating S-phase exit, cells that were treated with low-dose ER stress inducers were washed with a warm PBS, incubated with 20 µM of BrdU-containing media for 2 or up to 8 h, and immediately incubated with the 15 µM EdU-containing medium for 15 min. Cells were then harvested by trypsinization and cell suspension was centrifuged at 200× *g* for 5 min at 4 °C. Next, cells were resuspended in 1 mL of cold PBS, fixed with 2% paraformaldehyde at 37 °C for 10 min, centrifuged at 700× *g* for 5 min at 4 °C, and resuspended in ice-cold 90% methanol at room temperature for 30 min. After washing in a cold PBS, cells were stained with a 100 µL antibody dilution buffer (0.25% Tween-20-containing 1% BSA in a PBS) containing anti-BrdU mouse monoclonal (1:25, clone MoBU-1, B35141, Thermo Fisher Scientific, Waltham, MA, USA) for 1 h at room temperature. For experiments evaluating cell apoptosis, cells were stained with anticleaved Caspase 3 antibody (9661S, Cell Signaling, Danvers, MA, USA) or anti-Annexin V FITC-conjugated monoclonal antibodies (BMS147FI, VAA-33, eBioscience, San Diego, CA, USA) for 1 h at room temperature. For EdU detection, Alexa Fluor^TM^ 488 dye azide was conjugated to EdU via CuSO_4_-mediated Click chemistry reaction for 30 min at room temperature using Click-iT EdU Alexa Fluor^TM^ 488 dye Flow Cytometry Assay Kit (C10337, Thermo Fisher Scientific, Waltham, MA, USA; 250 µL reaction volume for one sample). For experiments evaluating the levels of cisplatin-DNA damage, cells were stained with anti-cisplatin modified DNA antibody (ab103261, Abcam, Cambridge, MA, USA). Cells were then washed with a cold PBS containing 1% FBS and resuspended in a 100 µL of antibody dilution buffer containing Alexa Fluor 647-conjugated donkey antimouse IgG (H + L) (1:800, A31571, Thermo Fisher Scientific, Waltham, MA, USA) for 30 min at room temperature. For samples that were stained with BrdU alone, cells were resuspended in a 200 µL of PBS containing 2 µg of propidium iodide (PI; P4170, Sigma-Aldrich, St. Louis, MI, USA) and 100 µg of RNase A (19101, Qiagen, Hilden, Germany). For experiments evaluating the levels of ROS, MitoSOX^TM^ Red Mitochondrial Superoxide Indicator (M36008, Thermo Fisher Scientific, Waltham, MA, USA) was used. Cells were washed twice, resuspended in a 200 µL PBS, and analyzed on a FACSCanto II Flow Cytometer (BD Biosciences), and the acquired data were analyzed using FlowJo version 9 (Tree Star). 

### 4.5. WST8 Assay

Cells that had been treated with 2.5 nM of thapsigargin or 1 µg/mL of tunicamycin for 4 days were harvested by trypsinization and seeded in a 96-well plate at the density of 7 × 10^3^ cells/100 μL/well for 18–24 h. Next, cells were washed with a PBS and incubated with medium containing 0 or up to 125 µM of cisplatin or 5-FU at 37 °C for 24 h. Cells were then washed with a warm PBS and resuspended in warm fresh media, and 10 µL of the Cell Counting Kit-8 reagent (CCK-8, #CK04, Dojindo Molecular Technologies, Kumamoto, Japan) was added in each well of the plate. After 4 h of incubation at 37 °C, absorbance was measured at 450 nm in a microplate reader. At least three independent replicate experiments were performed.

### 4.6. RNA Sequencing and Analyses

Library preparation and sequencing were performed at Genomics Inc. A standard nonstrand specific protocol with poly-A selection of mRNA was used. Briefly, Poly-T oligo-attached beads were used to purify mRNA from total RNA. The selected RNA was then heat fragmented and randomly primed for cDNA synthesis. The resultant cDNA then went through library preparation steps, including end repair, base A-tailing, multiple indexing adaptor ligation, enrichment, and PCR amplification. The prepared library was validated on an Agilent 2100 Bio-analyzer and Real-Time PCR System before proceeding to Illumina NextSeq sequencing. 

Paired-end sequencing data were cleaned by fastQC [89]. The FASTQ files of reads were processed by Salmon [90] (v1.0.0) under mapping-based mode using selective alignment to the reference transcriptome (Homo sapiens, GRCh38, GENCODE [91] release 29). The GC bias and position bias were corrected while running Salmon with flags “-gcBias” and “-posBias”. The gene-level read counts were put into GFOLD [92] (v1.1.4) to obtain generalized fold change for ranking differentially expressed genes. Expression values were normalized by the DESeq method with default option flag “-norm DESeq”. Genes with |GFOLD| > 0.1 were considered differentially expressed.

Gene Ontology (GO) analysis was performed to identify enriched GO annotations for genes that were differentially expressed. GO terms from Biological Process (BP), Cellular Component (CC), and Molecular Function (MF) categories were tested using GOseq [93] (v1.38.0). GOseq performs an overrepresentation test while accounting selection bias potentially existed in the RNA-seq data. Gene length data were obtained from the GTF file (Homo sapiens, GRCh38, Ensembl release 98) and were provided to GOseq for length bias correction.

Gene Set Enrichment Analysis [94] (GSEA) was performed to identify enriched functional gene sets in the RNA-seq experiment considering both strength and direction of differential expression signals between phenotypes. The ER stress adaptation was defined as the positive phenotype and the control as the negative phenotype. A collection of functional gene sets downloaded from the Molecular Signatures Database [95] (MSigDB) were screened for concordant differences between the phenotypes. The normalized enrichment score (NES) was used to rank gene set enrichment results. The sign of an NES indicates the direction of enrichment, and the absolute value of an NES reflects the strength of enrichment. A gene set with highly extreme (deviated from zero) NES implies that it is highly associated with the phenotype.

### 4.7. CRISPR-Mediated POLH Knockout

For the generation of a plasmid for clustered, regularly interspaced, short palindromic repeat RNA-guided (CRISPR) targeting POLH, we designed two sets of primers (5′-GTGATGAGTTACCATGAGCA-3′ and 5′-GGTGAGGTTAGCTT TCCCAC-3′) that separately direct Cas9 to the intron before the exon 2 and at the end of exon 3 of POLH. These sgRNAs were inserted into an all-in-one CRISPR vector, the pALL-Cas9.Ppuro (obtained from C6 RNAi core facility, Academia Sinica, Taiwan) with *BsmB*I digestion sites. This vector was used to transfect OECM1 by Lipofectamine 3000 Reagent (Thermo Fisher Scientific, Waltham, MA, USA) according to the manufacturer’s instructions. After 24 h, the medium was replaced with a fresh culture medium containing 2 µg/mL of puromycin for additional 3 days to select the transduced cells. Subsequently, a limiting dilution was used to establish single-cell clones. To verify the knockout of POLH, the genomic DNA of each clone extracted using a QIAamp^®^ DNA Mini kit (51304, QIAGEN) was used as the template for PCR amplification. The PCR reaction was performed in a 25 µL reaction mixture of KAPA HiFi DNA polymerase (Kapa Biosystems) with 1μg of genomic DNA and 0.3 μM of forward and reverse primers (5′-GCTCATGGTAACTCATCAGTG-3′ and 5′-GTTAGCTTTCCCACGGGAC-3′). A wild-type (916 bp) PCR amplicon spanning the target site was obtained following 32 cycles of amplification. Successful knockout of the gene segment by CRISPR hampered the generation of PCR products.

### 4.8. Immunofluorescence 

The cells that had been maintained in cell culture media with or without 2.5 nM of thapsigargin were trypsinized and seeded on 4-well chamber slides (154941, LAB-TEK) at a density of 3.5 × 10^4^ cells per well. After 18 to 24 h, cells were washed with a warm PBS and incubated in cell culture media containing 50 to 100 µM of Cisplatin-Texas Red conjugate for 24 h. Cells were then fixed with 4% paraformaldehyde for 10 min at room temperature, and subsequently permeabilized using 0.05% Trion-X100 in a PBS for 5 min. For TP53 staining, the antibody (175933, Abcam) was used. Cells on chamber slides were washed and the nuclei were counterstained with 4′,6-diamino-3-phenylidole, dihydrochloride (DAPI, #D1306, Thermo Fisher Scientific, Waltham, MA, USA) and examined using an Olympus FV1000 confocal microscope.

### 4.9. Genomic DNA Extraction and Targeted Sequencing

The genomic DNA of samples was extracted with a MagNA Pure Compact Nucleic Acid Isolation Kit (Roche). The target regions were amplified using KAPA HiFi HotStart PCR kit (KAPA Biosystems) in a total reaction volume of 50 µL. Reactions were run in a 9700 thermal cycler (Applied Biosystems) using the following cycling parameters: 3 min holding at 95 °C, followed by 25 cycles of 20 s at 95 °C, 20 s at 66 °C, and 30 s at 72 °C, and final hold at 4 °C. The presence of amplicons was confirmed by gel electrophoresis on a 1.5% agarose gel. The PCR amplicons were purified by using a PCR Fragment Extraction Kit (Geneaid, Taiwan). DNA sequencing was performed by using ABI PRISM BigDye Terminator Cycle Sequencing Ready Reaction Kit, v3.1 (Applied Biosystems) on the ABI PRISM 3730XL DNA Analyzer. The primer sequence was ex1-F, CTCCCCAACTCCATTTCCTT, ex1-R, GAAAATACACGGAGCCGAGA; ex2-F, TCAGACACTGGCATGGTGTT; ex3-R, GCCAGGCATTGAAGTCTCAT; ex4-F, CACTCTCAAAGAGGCCAAGG; ex5-R, CTTAACCCCTCCTCCCAGAG; ex6-F, CTTGGGCCTGTGTTATCTCC; ex7-F, TGCTAGGAAAGAGGCAAGGA; ex8-F, GCGCACAGAGGAAGAGAATC; ex9-R, GAATCGCTTGAACCCAGAAG; ex10-F, TGCATGTTGCTTTTGTACCG; ex11-R, CAAGGGTTCAAAGACCCAAA.

### 4.10. TP53 Knockdown 

The transfection for short hairpin (shRNA) to knockdown the TP53 was performed using Lipofamine 3000 (Thermo Fisher Scientific, Waltham, MA, USA) according to the manufacturer’s instruction. Two shRNA clones were obtained from the National C6 RNAi Core Facility at Academia Sinica in Taiwan. The target sequences were CGGCGCACAGAGGAAGAGAAT and CACCATCCACTACAACTACAT.

### 4.11. Statistics

The data in this study were presented as mean ± standard deviation. Statistical analyses of the Student’s *t*-tests and one-way ANOVAs were performed for two-group and multiple-group comparisons, respectively, using GraphPad Prism (version 5.0). Differences were considered statistically significant when *P* was < 0.05.

## Figures and Tables

**Table 1:** Clinico-pathological features of oral cancer patients with cisplatin-based chemotherapy prior to surgical treatment.

Patient ID.	Tumor Site	Tumor Staging	Radiographic Measurement before Treatment	Surgical Specimen Measurement after Treatment	% Change in Tumor Volume	GRP78 Pattern and Intensity	TP53 Exon	TP53 Mutation
1	Buccal	T4aN1M0	60 × 35 × 45 mm	65 × 45 × 40 mm	23.80%	Strong Diffuse	4 8	P72R E271K
2	Gingival	T4aN0M0	59 × 32 × 45 mm	50 × 35 × 42 mm	−13.49%	Moderate Diffuse	4	P72R
3	Buccal	T3N1M0	45 × 22 × 15 mm	45 × 30 × 10 mm	−9.09%	Strong Peripheral	4	P72R
4	Gingival	T4aN1M0	38 × 36 × 20 mm	43 × 25 × 25 mm	−1.77%	Moderate Peripheral	4	P72R
5	Tongue	T3N0M0	36 × 20 × 25 mm	38 × 30 × 20 mm	−9.52%	Moderate Diffuse	9	S315YS
6	Buccal	T4aN1M0	40 × 27 × 11 mm	36 × 22 × 14 mm	−6.67%	Moderate Peripheral	7	M237I
7	Tongue	T4aN0M0	38 × 33 × 27 mm	30 × 28 × 22 mm	−45.42%	Weak Peripheral	4 7	P72R R248Q

## Data Availability

Data is contained within the article and supplementary material. The data presented in this study are available online.

## Supplementary Materials

The following are available online at https://www.mdpi.com/1422-0067/22/1/355/s1.
