# Supplementary material for: Adaptation to Endoplasmic Reticulum Stress Enhances Resistance of Oral Cancer Cells to Cisplatin by Up-Regulating Polymerase η and Increasing DNA Repair Efficiency
Source: Int J Mol Sci. 2020 Dec 31;22(1):355. doi: 10.3390/ijms22010355 (PMC7794796; doi:10.3390/ijms22010355)

Supplementary Figure 1. Adaptation to ER stress enhances cisplatin resistance of SAS and HSC-3 cancer cell lines.

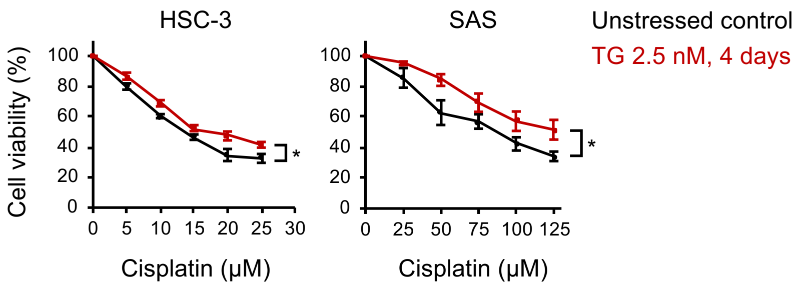

Supplementary Figure 2. Both S-phase cells and non-S phase cells acquire cisplatin resistance by adaptation to ER stress

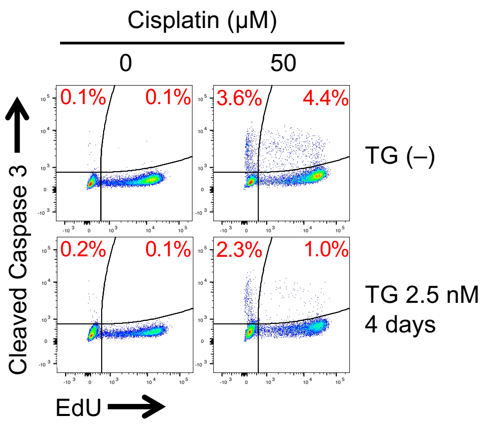

Supplementary Figure 3. Induction of *PUMA* and *NOXA* is not compromised in OECM1 cells harboring p53 missense

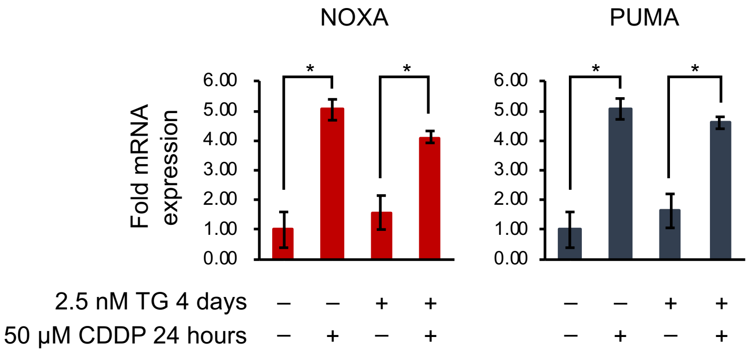

Supplementary Figure 4. 53BP1 translocates to nucleus in cells adaptive to ER stress

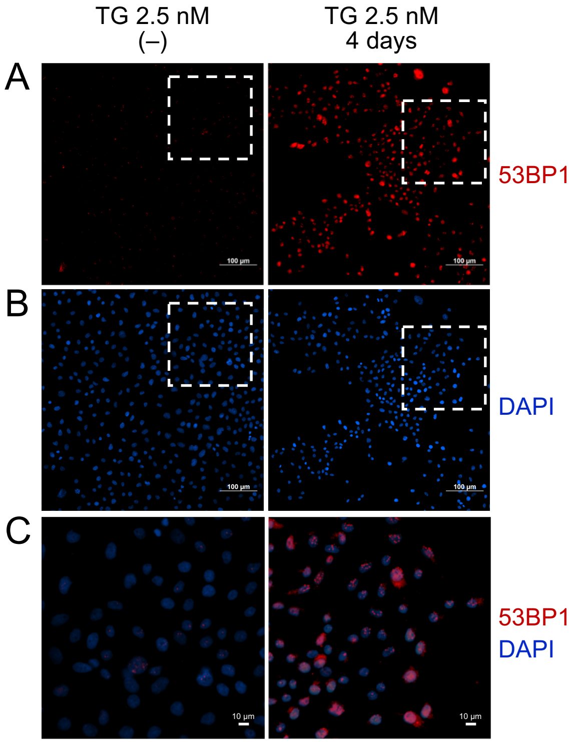

Supplementary Figure 5. Adaptation to persistent ER stress decreases the level of phospho-H2AX induced by cisplatin treatment.

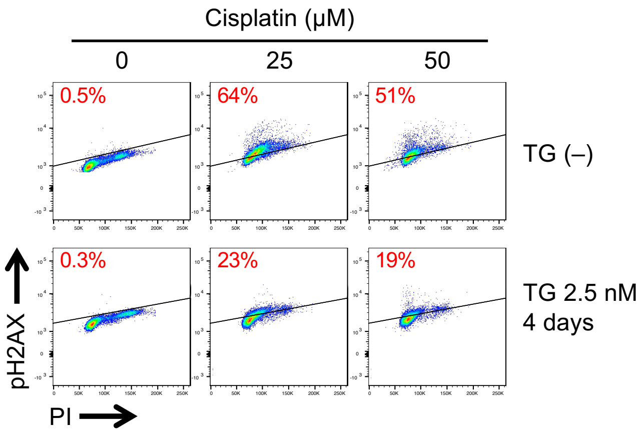

Supplementary Figure 6. The uptake of cisplatin was not affected in cells adaptive to ER stress

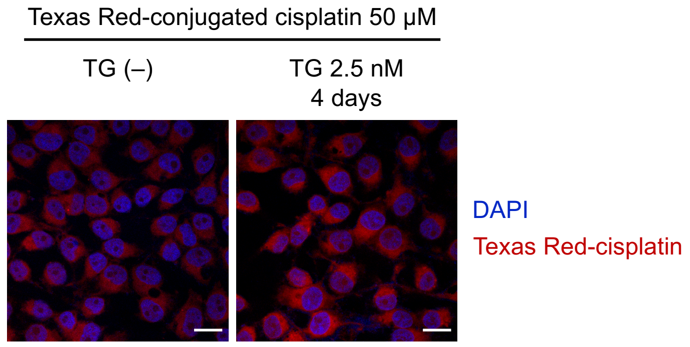

Supplementary Figure 7. Adaptation to persistent ER stress is accompanied by an increase in GADD45

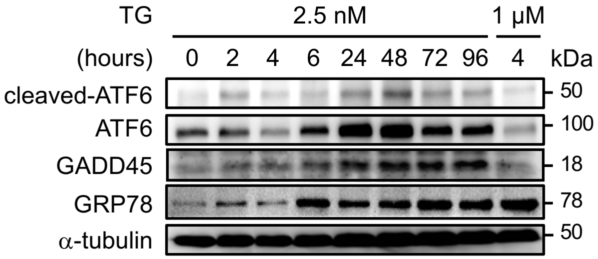

Supplementary Figure 8. Adaptation to ER stress lowers the level of intrinsic ROS but is unable to suppress the accumulation of cisplatin-induced ROS.

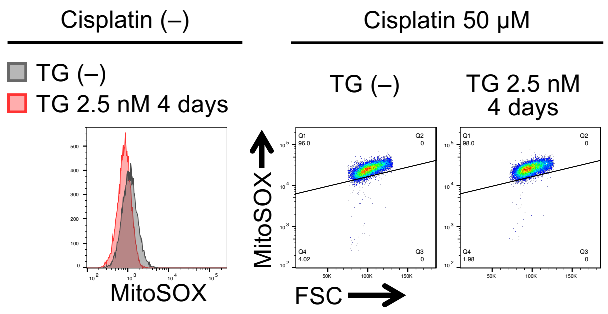

Supplementary Figure 9. P53-knockdown cancer cells are not sustainable under prolonged ER stress.

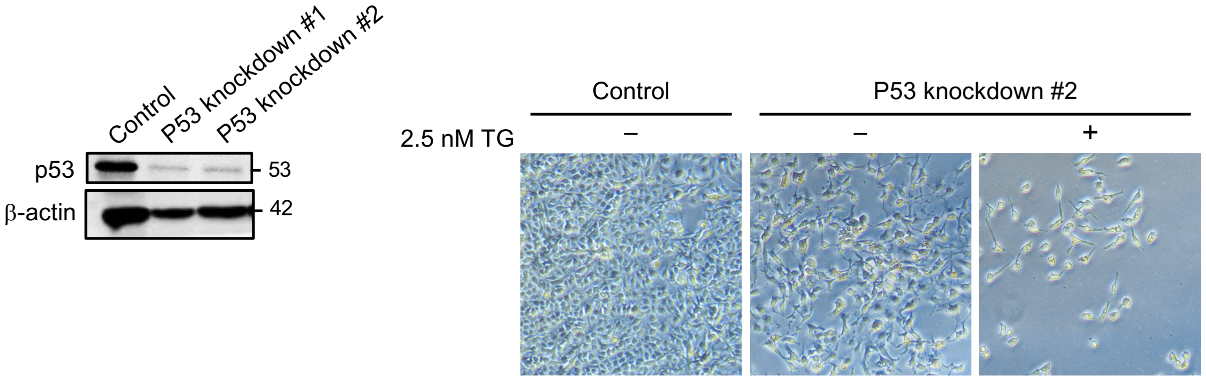

Supplement: Supplementary file 1 [file ijms-22-00355-s001.pdf]
